# Comparative Genomics Provides Insights into Adaptive Evolution in Tactile-Foraging Birds

**DOI:** 10.3390/genes13040678

**Published:** 2022-04-12

**Authors:** Li Wang, Li Sun, Qiu-Hong Wan, Sheng-Guo Fang

**Affiliations:** MOE Key Laboratory of Biosystems Homeostasis & Protection, State Conservation Centre for Gene Resources of Endangered Wildlife, College of Life Sciences, Zhejiang University, Hangzhou 310058, China; 21907093@zju.edu.cn (L.W.); sunli2012@zju.edu.cn (L.S.); qiuhongwan@zju.edu.cn (Q.-H.W.)

**Keywords:** tactile-foraging birds, trade-off, sensory systems, brain structures, adaptive evolution

## Abstract

Tactile-foraging birds have evolved an enlarged principal sensory nucleus (PrV) but smaller brain regions related to the visual system, which reflects the difference in sensory dependence. The “trade-off” may exist between different senses in tactile foragers, as well as between corresponding sensory-processing areas in the brain. We explored the mechanism underlying the adaptive evolution of sensory systems in three tactile foragers (kiwi, mallard, and crested ibis). The results showed that olfaction-related genes in kiwi and mallard and hearing-related genes in crested ibis were expanded, indicating they may also have sensitive olfaction or hearing, respectively. However, some genes required for visual development were positively selected or had convergent amino acid substitutions in all three tactile branches, and it seems to show the possibility of visual degradation. In addition, we may provide a new visual-degradation candidate gene *PDLIM1* who suffered dense convergent amino acid substitutions within the ZM domain. At last, two genes responsible for regulating the proliferation and differentiation of neuronal progenitor cells may play roles in determining the relative sizes of sensory areas in brain. This exploration offers insight into the relationship between specialized tactile-forging behavior and the evolution of sensory abilities and brain structures.

## 1. Introduction

The sense of touch is critical to detect environmental stimuli including pressures, vibrations, and textures of objects, which endows vertebrates with the ability of object recognition and discrimination and emotional transmission [1]. At times, a powerful sense of touch is necessary for organisms to hunt when they are less sensitive to vision or under conditions of poor visibility. To quickly and accurately capture tactile information, vertebrates have evolved specialized end-organs. For example, it has been reported that nocturnal mice and rats with poor vision rely on whiskers to explore the environment, such as textures and positions of objects, and apertures size [2,3,4,5]. Tentacles can help aquatic tentacled snakes forage in complete darkness [6], and similar functions are performed by the integumentary sensory organs of crocodilians [7] and the Eimer’s organs of star-nosed moles [8]. These end-organs are densely covered with mechanoreceptors, such as Meissner and Pacinian corpuscles in reptiles and mammals [9].

In birds, groups of Herbst or Grandry corpuscles are concentrated at the tip of bills, which allow birds to hunt or palpate depending solely on tactile clues [9,10]. According to different distribution patterns of mechanoreceptors on “bill-tip organs”, tactile specialists can be divided into three subtypes. (1) In probe-foraging birds, mechanoreceptors are embedded into bony pits (sensory pits) at the tip of their elongated bills. They probe for buried prey at a distance by detecting vibrations and pressures from water or sand, which is called “remote touch”. This probe-foraging strategy can be found in three phylogenetically separate families: kiwi of Apterygidae, ibises of Threskiornithidae, and shorebirds of Scolopacidae [11,12,13,14]. (2) In parrots, mechanoreceptors locate in touch papillae within keratinized rhamphotheca rather than bone, and they are arranged along the inner edges of the beaks. Therefore, the tactile clues in parrots are usually employed to manipulate objects, rather than find food [14,15]. (3) Waterfowl (ducks and geese) have mechanoreceptors organized on both the inner and outer surfaces of the bills, including touch papillae in the rhamphotheca and tongue, and sensory pits in the bone. The strategy adopted by waterfowl is dabbling and they can search for food concealed in mud [16,17]. In these species, tactile information detected by these mechanoreceptors are transmitted from trigeminal afferents to the principal sensory nucleus (PrV) in the brain for further processing [18,19,20]. Although they have different facial sensation regions, significantly enlarged PrVs are shared among the three subtypes of tactile-specialized birds [14,21].

It is worth noting that several birds of the palaeognaths also possess bony “bill-tip organs” that are similar to probing-foraging birds; however, they neither depend on tactile information to forage nor have enlarged PrVs [22,23,24]. For example, though equipped with bony “bill-tip organs”, the ostrich is one of the vertebrates with the largest eyes and is visually guided when accurately pecking for food [25,26]. One possible explanation has been proposed that the bony “bill-tip organs” across these species originate from a common ancestor in the cretaceous period [10]. What causes the divergence of foraging strategies between birds with similar tactile end-organs that may be inherited from the common ancestor? For what reason is the tactile-foraging behavior not lost in probing birds and dabbling waterfowl? Is the reason that other senses are less sensitive or have suffered degradation in tactile foragers? Or to improve competitiveness in niches they occupied, whether tactile-foraging behavior is adopted with reduced reliance on vision or olfaction? The relative sizes of neural structures responsible for specific behaviors are positively related with the complexity and amount of processing from inputs, which are largely independent of phylogeny in vertebrates [27]. Compared to visually guided birds, the hypertrophy of PrVs but smaller brain regions (such as optic tectum and nucleus rotundus) related to visual pathways were found in waterfowl, beak-probing shorebirds, and kiwi [28,29]. It seems to mean that the PrVs are expanded at the expense of the reduction of visual-processing areas in tactile-foraging birds. The phenomenon of “trade-off” can also be found in studies of ducks with different degrees of tactile dependence, and the results showed that there were more mechanoreceptors but fewer thermosensors and nociceptors in tactile-foraging ducks [30,31]. In addition, foraging strategies contribute to regional specialization of retinal ganglion cells, and are associated with different types of visual fields in avians [32,33,34,35]. This evidence seems to indicate that the specialized tactile-foraging behavior may be associated with the evolution of sensory abilities and brain structures. At the same time, “trade-off” may exist between different senses, as well as between different sensory-processing areas in brain.

Compared to the rapidly increasing studies of other senses, less attention has been paid on tactile perception. Although there are some studies that have focused on the morphological comparison of “bill-tip organs” or PrVs, the neuronal mechanism of mechanosensitivity, as well as foraging behaviour observations, the molecular basis of the relationship between specialized tactile-forging behavior, and the evolution of sensory abilities and brain structures have not yet been clarified. Therefore, we attempted to explore the mechanism underlying the evolution of sensory systems in tactile-foraging birds at the genomic level. In this study, three tactile-foraging species were selected as representatives from different orders: crested ibis (*Nipponia nippon*) [36] of Pelecaniformes, mallard (*Anas platyrhynchos*) of Anseriformes, and northern brown kiwi (*Apteryx mantelli*) of Apterygiformes. Each of their non-tactile sister species was set as a control: little egret (*Egretta garzetta*), chicken (*Gallus gallus*), and white-throated tinamou (*Tinamus guttatus*), respectively. Finally, the visual-foraging common ostrich (*Struthio camelus*) was chosen as the outgroup. The relationships refer to the widely accepted phylogeny [37]. This exploration may provide a reference and offer insights into the evolutionary specialization in other tactile-specialized vertebrates. 

## 2. Materials and Methods

### 2.1. Data Collection

Whole-genome sequences of all seven species were collected from NCBI (http://www.ncbi.nlm.nih.gov, accessed on 21 January 2021): crested ibis (*N. nippon*, GCF_000708225.1), mallard (*A. platyrhynchos*, GCF_003850225.1), northern brown kiwi (*A. mantelli*, GCF_001039765.1), little egret (*E. garzetta*, GCF_000687185.1), chicken (*G. gallus*, GCF_000002315.6), white-throated tinamou (*T. guttatus*, GCF_000705375.1), and the outgroup common ostrich (*S. camelus*, GCF_000698965.1). Genes with less than 30 amino acids and stop codons, were removed. At the same time, we only extracted the longest transcript for each species when multiple transcripts of one gene.

### 2.2. Identification of Orthologues and Phylogenetic Tree Construction

We obtained the similarity relationships between protein sequences of all species through Blastall 2.2.26, with an e-value of 1 × 10^−5^. OrthoMCL 1.4 [38] was chosen to cluster the protein alignments with an inflation of 1.5. For each 1:1 single-copy gene family, sequences were aligned using MUSCLE 3.8.31 [39]. After merging all selected alignments, the super alignment matrix was generated and used to construct a maximum likelihood tree with RAxML 8.2.12 [40]. The divergence time was estimated using r8s 1.81 [41] with the default parameters; results with time calibration points taken from the TimeTree website were used as the input to mcmctree 4.9 [42].

### 2.3. Gene Family Expansion and Contraction

During evolution, the changed core families may play an important role in forming a special adaptation mechanism. To identify the expanded or contracted gene families related to tactile-foraging phenotypes, CAFE 4.2 [43] was adopted to estimate the evolution of gene family sizes based on two inputs: the time tree constructed above and the gene family sizes resulting from orthologues clustering. The number of significantly changed gene families (Family-wide *p*-value < 0.05, and Viterbi *p*-values < 0.05) was identified on each branch of the ultrametric tree.

### 2.4. Positive Selection Analysis

Adaptive evolution results from natural selection, and changes in selective pressures, may lead to different directions of evolution. To detect genes positively selected in the tactile-foraging group (TG), they were considered common foreground branches; other species were divided into the background group (BG). Single-copy gene families that were aligned were set as templates to generate corresponding cds alignments using a script. With cds alignments, the branch-site model of codeml in the PAML 4.9 [42] was performed, and genes were seen as positively selected using the likelihood ratio test (*p* < 0.05). At the same time, the average *dn*/*ds* (ω) values of all single-copy gene families in TG and BG birds were obtained, and the difference values (ω(TG)−ω(BG)) were calculated to identify the direction of selection. Genes with significantly different *dn*/*ds* (ω) were focused (*p* < 0.01) [44].

### 2.5. Identification of Convergent Amino Acid Substitutions

Distantly distributed tactile-foraging birds have developed a series of specialized sensory phenotypes, so the method of identifying convergence at conservative sites (CCS) was applied to detect adaptive convergence signals [45]. Orthologues were identified using the OrthoFinder 2.3.11 [46], and sequences were aligned using PRANK v.170427. Trimal v1.4.rev.22 was used to remove the gaps in sequence alignments. JTT + γ amino acid substitution model of evolver in PAML was used to infer the amino acids status of the ancestor in each node, and characters with the highest posterior probabilities were saved. The simulated status were compared to the outgroup to ensure the accuracy [47]. Convergent sites were identified with the following criteria: (1) tactile-foraging birds (crested ibis, mallard, and northern brown kiwi) shared the same characteristics; (2) the characteristics of non-tactile foraging birds (little egret, chicken, and white-throated tinamou) and ancestors were identical; (3) the amino acid residues between the TG and BG birds were different. These strict conditions greatly reduce random convergence and false inference of the ancestral character.

## 3. Results

### 3.1. Identification of Orthologues and Phylogenetic Tree Construction

**Figure 1:**
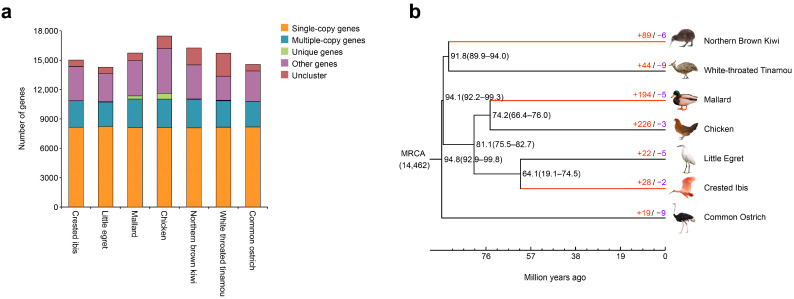
(**a**) Gene families clustering results of all species. (**b**) Time tree construction, and expansions and contractions of gene families of each species. Black numbers at nodes were divergence time between related branches, and 95% confidence intervals were followed (in parentheses). Three tactile-foraging branches were in red, and the number of genes expansions (+, red) and contractions (−, purple) were also shown on each branch.

According to the results of clustering, 14,462 gene families were obtained (Table S1). They can be divided into five categories in each species: single-copy genes, multiple-copy genes, unique genes, other genes, and unclustered genes (Figure 1a). Overall, the chicken had the largest number of genes, and the little egret had the fewest. However, the proportion of each type was roughly equal among these species, and there were more unique genes in mallard and chicken than in others. Moreover, single-copy genes accounted for almost half of the genes in each species. Using a total of 7648 single-copy orthologous genes, we successfully constructed a phylogenetic tree. Subsequently, the divergence time between species was obtained, and the phylogenetic tree was converted to an ultrametric tree (Figure 1b).

### 3.2. Gene Family Analysis

The gain or loss of genes was obtained for all birds by simulating the evolution of gene families in CAFE. The numbers of expanded and contracted gene families are shown on each branch (Figure 1b). In general, there were more expanded genes for each species compared to the most recent common ancestor. In total, 89, 194, and 28 gene families were expanded in kiwi, mallard, and crested ibis, respectively. Gene ontology (GO) and KEGG enriched analyses were performed for these expanded genes, and most of them were mainly related to signal transduction, metabolism, and immune response (*q* < 0.05). Some expanded genes were enriched in pathways associated with neural development in three tactile foragers. For example, the “MAPK signaling pathway” (ko04010) and the “mTOR signaling pathway” (ko04150) shared in crested ibis and mallard can play roles in regulating neural progenitor cells proliferation and dendrite development in the process of neurogenesis [48,49]. The “TGF-ß signaling pathway” (ko04350) enriched in mallard is associated with neurite growth, and proliferation and migration of neural cells [50]. What’s more, GO terms related to the nervous system were also identified (*q* < 0.05), including “central nervous system development” (GO:0007417), “peripheral nervous system development” (GO:0007422), “negative regulation of axon extension involved in axon guidance” (GO:0048843), and “positive regulation of neuron death” (GO:1901216) in kiwi, and “regulation of neuron differentiation” (GO:0045664), “dendritic cell migration” (GO:0036336), “response to axon injury” (GO:0048678), “negative regulation of neuron death” (GO:1901215), and “neuron apoptotic process” (GO:0051402) in crested ibis (Figure S1, Table S2). At the same time, expanded gene families showed the relations with different sensory systems in three tactile foragers. In kiwi, the four enriched GO terms were associated with olfactory system, which was consistent with the results that they had a higher diversity of olfactory receptors compared to other birds. Acute olfaction was necessary for kiwi since they mainly depend on tactile and olfactory clues to forage [51,52]. In mallard, expanded genes were enriched in the term of “olfactory receptor activity”, and it may be associated with olfactory receptor family 14 whose expansion had been reported [53]. In crested ibis, two enriched GO terms were associated with the development of auditory organ (“stereocilium tip” and “inner ear development”), and one term was involved in auditory perception (Table 1).

### 3.3. Positive Selection Analysis

**Figure 2:**
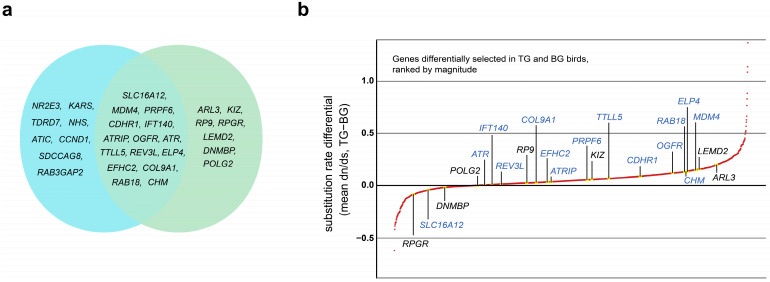
(**a**) The Venn diagram shows visual-degradation candidate genes identified in positive selection analysis; 23 genes in the blue circle were positively selected in tactile-foraging birds, and 22 genes suffered significantly different selective pressures between the tactile-foraging group (TG) and the background group (BG) birds. (**b**) All genes with significantly different selective pressures (nonsynonymous to synonymous substitution ratios (*dn*/*ds* or ω)) between TG and BG birds were marked in red. A subset of 22 visual-degradation candidate genes were marked in yellow, and 15 sharing genes were also highlighted in blue.

To determine the molecular basis of adaptive evolution in tactile-foraging birds, positive selection analysis was performed. Finally, we obtained 1025 genes that were positively selected in TG birds, and 1807 genes with significantly different *dn*/*ds* values between the TG and BG birds, respectively. Functional enrichment analysis showed that these genes mainly participated in metabolism, genetic information processing, and cellular processes (Table S3). Indeed, 23 positively selected genes in TG birds and 22 differently selected genes between TG and BG birds were found to be essential for the normal development of vision. In addition, there are 15 visually-related genes shared in the two pipelines (Figure 2a). *CDHR1*, *TTLL5*, *PRPF6*, and *IFT140* have been reported to play roles in human disorders of cone-rod dystrophy and cone dystrophy or retinitis pigmentosa [54,55,56,57]. The polymorphisms of *MDM4* and increased expression of MDM4 protein promote retinoblastoma in mice and human [58,59]. The mutation or deletion of *ELP4* can suppress the expression *PAX6* which controls eye development, and results in aniridia syndrome characterized by impaired vision and nystagmus [60]. Choroideremia is clinically characterized by progressive vision loss with the degeneration of choriocapillaris and retinal pigment epithelium, resulting from mutations of the *CHM* gene [61]. *RAB18* has been proven to have a role in regulating neuronal migration, and the mutations of *RAB18* led to Warburg-Micro syndrome, whose classic phenotype is abnormalities of the brain and eye [62,63]. *OGFR* contributes to the homeostasis of the cornea and has been linked to keratopathy [64]. The ATRIP–ATR complex mainly plays important roles in the process of retinogenesis and maintaining retinal integrity, and the inactivation of *ATRIP* in retinal progenitor cells causes the loss of vision in mouse [65]. The structure strength of vitreous was mainly maintained by collagen types Ⅱ and Ⅺ, and *COL9A1* was demonstrated to be key to the structural association of the two collagen types. The patients with mutations in *COL9A1* were characterized by vitreous degeneration [66]. The contiguous deletion containing *EFHC2* gene results in congenital blindness, and *REV3L* was one of candidate genes who can impair the activity of abducens nerves that endow eyes the ability of looking to the side [67,68]. Except for these genes involved in inherited diseases, mutations of *SLC16A12* may function in age-related cataracts through influencing lens homeostasis [69]. 

We ranked all genes that were differently selected in TG and BG birds. A subset of 22 visual-degradation candidate genes were annotated. (Figure 2b). Apart from the 15 highlighted genes described above, mutations of *ARL3*, *KIZ*, *RP9*, and *RPGR* were involved in retinitis pigmentosa [70,71,72,73], *LEMD2* and *DNMBP* were involved in juvenile cataracts or infantile cataracts [74,75], and *POLG2* was involved in progressive external ophthalmoplegia [76]. The results showed that most visual-degradation candidate genes had greater ω in TG birds, indicating that genes essential for visual-development were more likely to mutate in TG birds. At the same time, several other genes we identified were pathogenic in some congenital malformation syndromes with ocular defect, including Bardet–Biedl syndrome and Joubert syndrome. Both of the two syndromes are caused by the dysfunction of primary cilia which play important roles in signal transduction, such as detecting light, mechanical and biological signals. Patients are usually characterized by retinal degeneration, brain and limbs anomalies, liver or kidney dysfunction, and polydactyly. In Bardet–Biedl syndrome, *TTC8*, *BBS9*, *WDPCP*, and *IFT27* were shared by the two pipelines, and *BBS4*, *BBS7*, *BBS12*, and *IFT74* suffered significantly different selective pressures between TG and BG birds. The mutations of these genes hindered proteins transportation and location in primary cilia [77,78,79,80,81]. Half of these genes had greater ω in TG birds (*TTC8*, *WDPCP*, *IFT27*, and *IFT74*). Four positively selected genes that were involved in Joubert syndrome also had effects on cilium biogenesis and ciliary signaling. The genetic variants of *TCTN3*, *CC2D2A*, *TMEM231*, and *TCTN2* were related to the clinical subgroups with nystagmus or retinopathy [82,83]. At the same time, *ZNF335*, which can regulate the proliferation and differentiation of neuronal progenitor cells and may be responsible for the brain size, was positively selected and had greater ω in TG birds [84,85] (Table S3). 

### 3.4. Identification of Convergent Amino Acid Substitutions

Based on the principle of the CCS method, we explored whether there was convergent evidence among tactile-foraging birds, and 104 genes with convergent amino acid were identified. To filter false-positive signals, the groups of TG and BG were abolished, and three of the seven species were randomly chosen to detect convergent signals (crested ibis, chicken, and white-throated tinamou). Then, we obtained 256 genes with convergent signals. Although there were 10 genes which appeared again, the loci where convergent substitutions occurred were different in the two analyses, which verified the reliability of the results (Table S4D,E). Functional enrichment analysis of the 104 genes indicated that they were significantly enriched in metabolism and environmental information processing (Table S4).

**Figure 3:**
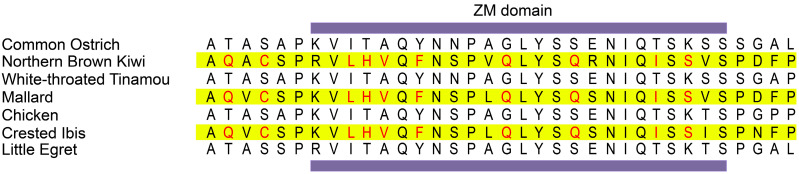
The convergent amino acid substitution sites of *PDLIM1* in tactile-foraging birds.

Similar to *ZNF335* identified in positive selection analysis, *ANKLE2*, which shared convergent substitutions in tactile-foraging birds, was required for the neuroblast proliferation and regulates brain development in *Drosophila* [86]. In addition, we obtained three genes that were related to human disorders with eye anomalies. Numbers of mutations or large deletions of *FRAS1* lead to the dermal-epidermal detachment, which are responsible for Fraser syndrome with cryptophthalmos characterized by eyelid anomalies [87,88]. The enriched GO terms with *FRAS1* involved contained “cell–cell adhesion” (GO:0016337), “cell adhesion” (GO:0007155), and “biological adhesion” (GO:0022610) (*p* < 0.05, Table S4). These biological processes are associated with structural support of tissues, cell proliferation, adhesion and morphogenesis during embryonic development in mammals. *DNA2* may play roles in mtDNA replication, and the deletions of mtDNA lead to progressive external ophthalmoplegia [89]. The third gene we identified was *MKKS*, and the mutations had been detected in patients of Bardet–Biedl syndrome [90]. In addition, *PDLIM1*, who suffered dense convergent amino acid substitutions within the ZM domain, plays an important role in the development of neurons (Figure 3). As an actin cytoskeletal protein widely distributed in multiple tissues, PDLIM1 controls neurite outgrowth and functions in rearrangement of the actin cytoskeleton during nerve regeneration in the peripheral nervous system [91,92]. In the central nervous system, a recent study of chicken retina showed that PDLIM1 proteins are expressed at the photoreceptors synaptic terminals, indicating that they may be involved in endocytosis or exocytosis during visual signals transmission, and the cytoskeleton arrangement may determine structures of photoreceptor ribbon synapses [93].

## 4. Discussion

Active tactile perception is critical for vertebrates to survival, especially when other senses are less sensitive. The evolutionary specialization of sense is often accompanied by the relative increased sizes of processing areas in the brain, which is called “principle of proper mass”. Additionally, the development of one sensory structure is also associated with other sensory structures, and there is usually a “trade-off” between the relative sizes of different sensory systems [27]. For example, the star-nosed mole has suffered regressive evolution of the visual system after it entered the subterranean environment, but the 22 tactile appendages around their nostrils make it the fastest known predator in mammals [94,95]. Correspondingly, there are reduced visual systems but an expanded trigeminal system in the star-nosed mole [96]. Compared to the normal group, there is evidence that congenitally blind cats have longer and more vibrissae to collect tactile information and improved auditory abilities, which ensure that the behaviors are only slightly impaired [97]. Echolocating bats have evolved expanded auditory structures but reduced visual structure of tectum opticum [98]. Considerable evidence has been found to support the “trade-off” in many vertebrates, including tactile-foraging birds. During adaptation to the new habitat of ground-dwelling caves, kiwi have suffered from the inactivation of several opsin genes, which is associated with the development of nocturnal-type retina. At the same time, the relative smaller eyes and fewer optic lobes compared to other nocturnal birds, and the relatively smaller brain regions processing visual information indicate the reduced reliance on vision [28,52,99]. Similarly, smaller sizes of the isthmo optic nucleus, optic tectum, nucleus rotundus, and entopallium, which are related to visual pathways, were found in waterfowl and beak-probing shorebirds [29,100]. With three tactile-foraging birds selected, we tried to explore the mechanism underlying the specialized evolution of sensory systems at the genomic level.

The results showed that expanded genes were enriched in olfactory systems, indicating kiwi and mallard may be relatively good at olfaction, which supported the conclusions of previous studies [51,52,53]. Differently, crested ibis may have sensitive hearing. To our knowledge, the auditory systems of crested ibis were relatively rarely studied, and therefore we can provide some new insight. However, several genes whose mutations or deletions may impair vision were positively selected (*CDHR1*, *TTLL5*, *PRPF6*, *IFT140*, *MDM4*, *ELP4*, *CHM*, *RAB18*, *OGFR*, *ATRIP*, *ATR*, *COL9A1*, *EFHC2*, *REV3L*, and *SLC16A12*), or had convergent amino acid substitutions (*FRAS1*, *DNA2*, *MKKS*) in all three tactile foragers, indicating the possibility of less-sensitive visual systems. In addition, some genes who had greater ω in TG birds were responsible for signaling and structure support in cells of vertebrates (*TTC8*, *BBS9*, *WDPCP*, and *IFT27*). They have been associated with human disorders, and the syndromes are usually characterized by vision abnormalities. On the other hand, *PDLIM1*, which had dense convergent amino acid substitutions, has been proven playing roles in chicken retina. However, it is not clear how many substitutions will function on the protein function. Attempts to predict the 3D structure using the SWISS-MODEL were unsuccessful during the course of this study; therefore, determining the function of *PDLIM1* and the ZM domain in visual system should be the focus of future work.

In addition, there were several genes and enriched pathways may be associated with the sizes of sensory processing areas. Recent studies have shown that the mechanisms of the increasing volume of the brain are usually various in different vertebrates. Generally, it is mainly influenced by two factors: the numbers and morphologies of neurons, such as relatively more neurons, or fewer but larger neurons. For example, there are decreased numbers but expanded sizes of neurons in the auditory system of galliforms, and a similar rule is applied in primate visual structures [101,102]. However, the relatively bigger brain of chicken compared to quail is the result of faster cell cycle rates before neurogenesis, and the proportionately expanded telencephalon in parrots may be related to delayed telencephalic neurogenesis. Faster cell cycle rates or delayed telencephalic neurogenesis both contribute to the proliferation of neuronal precursor cells [103,104]. Functional analysis of expanded gene families in tactile foragers showed that the “MAPK signaling pathway”, the “mTOR signaling pathway”, the “TGF-ß signaling pathway”, and several GO terms related to neuronal development were enriched, indicating their roles in regulating neural progenitor cell proliferation or dendrite development in the process of neurogenesis [48,49,50]. *ZNF335* and *ANKLE2* are responsible for regulating the proliferation and differentiation of neuronal progenitor cells, and both are candidate genes of autosomal recessive primary microcephaly, which is characterized by reduced skull circumference and brain volume [84,85,86]. Our results suggested that there were several signaling pathways, and two genes may play roles in regulating brain sizes, but it was not clear if they were involved in the reduction of visual processing areas or the expansion of PrVs. If they underlie the molecular basis of enlarged PrVs, it seems to indicate that there are fewer but larger neurons. The evidence of mechanoreceptive neurons with larger diameters in trigeminal ganglia allow us to propose this suggestion [30]. The exact mechanism needs to be explored in the future. 

Compared to visual-foraging birds, tactile-foraging ducks had a proportionally expanded number of mechanoreceptors in the trigeminal ganglia and expression of Piezo2. However, it is difficult to observe this trend at the genome level. There are mainly two difficulties, (1) mechanoreceptors located in other body parts still play important roles in both tactile and non-tactile specialists, such as Herbst corpuscles in wings, which can adjust flight behaviour by detecting vibrations of air currents, and corpuscles in legs that may be related to abnormal bird behaviour before an earthquake [105,106,107]. In mammals, Piezo2 is also expressed in the lung, small intestine, and dorsal root ganglia, which mediates function such as normal breathing, pain, and somatesthesia [108,109,110]. (2) Compared to other senses, the complex tactile-foraging behaviour is far from well understood in birds, and experiments on more species need to be conducted.

The limitation of the results found in this study can be due to several factors. For example, only three tactile-foraging species were included in this study due to the limitation of available genomes. Considering the CCS method, no other species were selected other than three sister species. This was to ensure that we only investigated species with clearly defined relationships. In addition, if the analysis of expanded gene families in the sister species was performed, the relations between pathways and phenotypes will be stronger. At last, although it can drastically reduce false positives, the CCS method is relatively conservative, and we acknowledge that our study may be introducing some false-negative signals. The CCS method was chosen as we wanted to be confident in sites that were identified, but more comprehensive methods should be explored in subsequent studies.

## 5. Conclusions

The foraging behavior is associated with adaptive evolution of sensory abilities and brain structures in birds. The “trade-off” may exist between different sense abilities in tactile foragers, as well as between different sensory-processing areas in the brain. We explored the mechanism underlying the specialized evolution in three tactile-foraging birds (kiwi, mallard, and crested ibis) at the genomic level. The results showed that expanded genes were significantly enriched in GO terms of olfactory systems in kiwi and mallard, and the enriched terms in crested ibis were associated with the hearing system, indicating that they may also have sensitive olfaction or hearing, respectively. However, it seems that they may have all suffered visual degradation. The reason is that some genes required for visual development were positively selected or had convergent amino acid substitutions in all three tactile branches, and mutations or deletions of these genes have been linked to visual impairment in mammals. In addition, we may provide a new visual-degradation candidate gene *PDLIM1* who suffered dense convergent amino acid substitutions within the ZM domain. At last, there were two genes responsible for regulating the proliferation and differentiation of neuronal progenitor cells that may play roles in regulating the sizes of brain areas processing sensory inputs. Our findings may help better understand the physiological processes and abnormal foraging behaviors in birds, especially for the endangered crested ibis. In addition, we provide some evidence regarding the relationship between specialized behavior and development of sensory systems. This is only a preliminary study of adaptive evolution in tactile foragers. More tactile specialists should be involved, and more comprehensive methods should be performed in further studies.

## Figures and Tables

**Table 1:** Gene ontology (GO) terms related to the development of sensory systems in expanded genes of tactile foragers.

Species	ID	Term	Category	FDR (<0.05)
Northern brown kiwi	GO:0004984	olfactory receptor activity	Molecular Function	5.59 × 10^−^^9^
	GO:0005549	odorant binding	Molecular Function	9.52 × 10^−^^3^
	GO:0050911	detection of chemical stimulus involved in sensory	Biological Process	5.59 × 10^−^^9^
	GO:0007608	sensory perception of smell	Biological Process	3.66 × 10^−^^2^
Mallard	GO:0004984	olfactory receptor activity	Molecular Function	2.29 × 10^−71^
Crested ibis	GO:0032426	stereocilium tip	Cellular Component	1.07 × 10^−^^3^
	GO:0048839	inner ear development	Biological Process	3.94 × 10^−^^3^
	GO:0007605	sensory perception of sound	Biological Process	2.60 × 10^−^^2^

## Data Availability

Not applicable.

## Supplementary Materials

The following are available online at https://www.mdpi.com/article/10.3390/genes13040678/s1, Table S1: Gene family clustering data for all species; Table S2 and Figure S1: Results of functional enrichment analysis for expanded or contracted gene families in three target species; Table S3: Positively selected genes and genes with significantly different selective pressures in the two groups and functional enrichment analysis; Table S4: Sites of convergent amino acid substitutions and functional enrichment analyses.
