# Supplementary material for: Comparative Genomics Provides Insights into Adaptive Evolution in Tactile-Foraging Birds
Source: Genes (Basel). 2022 Apr 12;13(4):678. doi: 10.3390/genes13040678 (PMC9028243; doi:10.3390/genes13040678)
Supplement: Supplementary file 1 [file genes-13-00678-s001.zip › Figure S1.pdf]

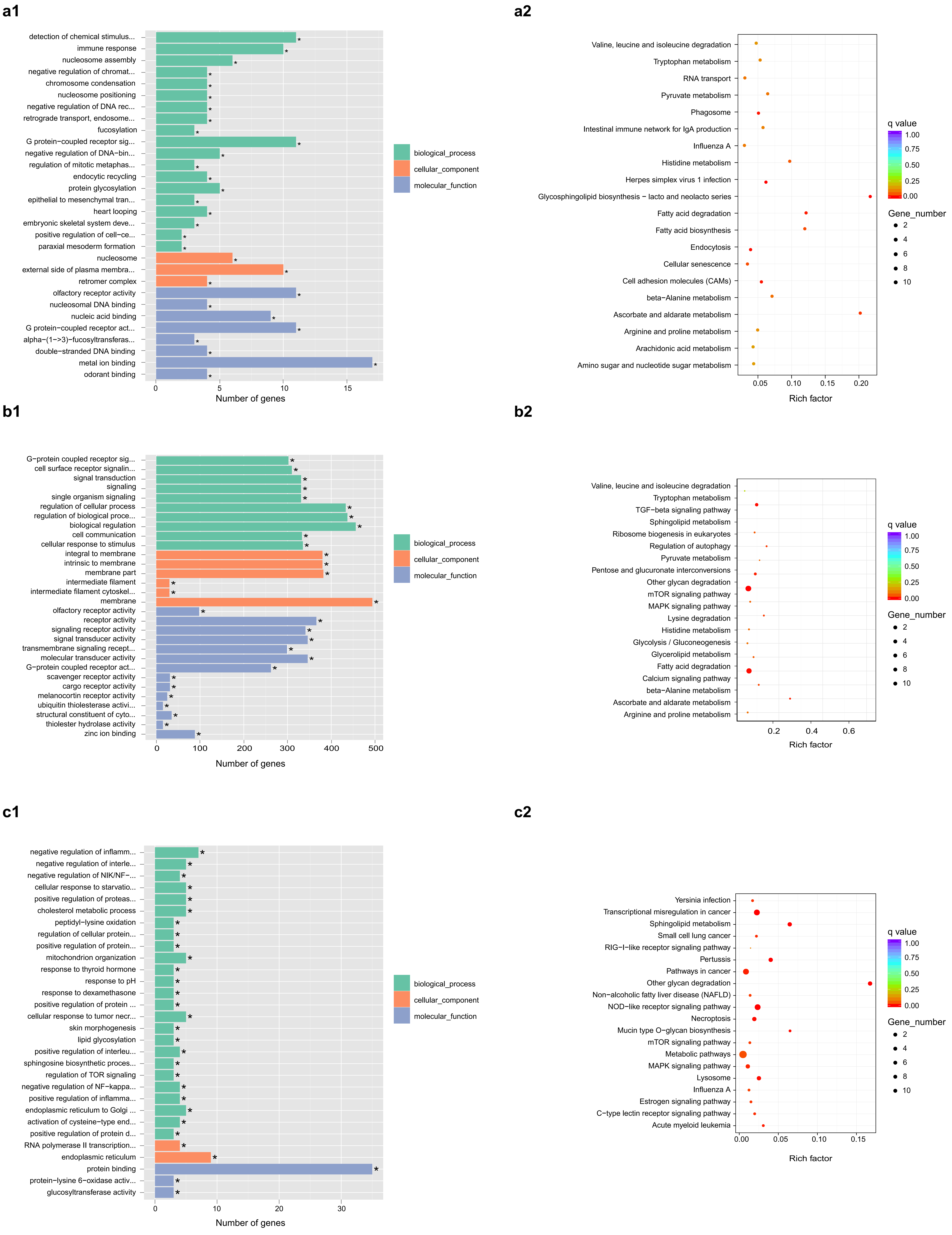

Figure S1. Reuslts of functonial enrichment analysis for expanded gene families in three target species. (**a**) Northern brown kiwi, *a1*: GO terms, *a2***:** KEGG pathways. (**b**) Mallard, *b1*: GO terms, *b2*: KEGG pathways. (**c**) Crested ibis, *c1*: GO terms, *c2*: KEGG pathways.
